# Complete Genome Sequence of *Spiroplasma turonicum* Tab4c^T^, a Bacterium Isolated from Horse Flies (*Haematopota* sp.)

**DOI:** 10.1128/genomeA.01010-16

**Published:** 2016-09-22

**Authors:** Wen-Sui Lo, Gail E. Gasparich, Chih-Horng Kuo

**Affiliations:** a Institute of Plant and Microbial Biology, Academia Sinica, Taipei, Taiwan; b Molecular and Biological Agricultural Sciences Program, Taiwan International Graduate Program, National Chung Hsing University and Academia Sinica, Taipei, Taiwan; c Graduate Institute of Biotechnology, National Chung Hsing University, Taichung, Taiwan; d Department of Biological Sciences, Towson University, Towson, Maryland, USA; e College of Arts and Sciences, Salem State University, Salem, Massachusetts, USA; f Biotechnology Center, National Chung Hsing University, Taichung, Taiwan

## Abstract

*Spiroplasma turonicum* Tab4c^T^ was isolated from a horse fly (*Haematopota* sp.; probably *Haematopota pluvialis*) collected at Champchevrier, Indre-et-Loire, Touraine, France, in 1991. Here, we report the complete genome sequence of this bacterium to facilitate the investigation of its biology and the comparative genomics among *Spiroplasma* spp.

## GENOME ANNOUNCEMENT

*Spiroplasma turonicum* is a bacterium associated with *Haematopota* sp. horse flies. The type strain Tab4c^T^ was isolated from a single fly, probably *Haematopota pluvialis*, collected at Champchevrier (Indre-et-Loire, Touraine, France) in 1991 and was assigned to group XVII within the genus (1). As part of our ongoing effort to investigate *Spiroplasma* genome evolution (2), we determined the complete genome sequence of *S. turonicum* Tab4c^T^.

The DNA sample was prepared from the strain maintained in Gail Gasparich’s laboratory at Towson University, which was acquired from the USDA/ARS *Spiroplasma* Culture Collection of Robert Whitcomb in 1996. This strain had been lyophilized after 17 passes from the original isolation. Prior to our completion of this project, a complete genome sequence of this bacterium was published based on another subculture of the same strain using the Pacific Biosciences platform (3). Because of this, we utilized this published sequence (GenBank accession no. CP012328.1) as the reference for a resequencing analysis. We chose the Illumina MiSeq platform to generate 301-bp reads from one paired-end library (~510-bp insert, 1,206,242 reads, ~288-fold coverage). The raw reads were mapped to the reference genome using BWA version 0.7.12 (4), programmatically checked using SAMTOOLS version 1.2 (5), and visually inspected using IGV version 2.3.57 (6).

The procedures for genome annotation were based on those described in our previous studies on *Spiroplasma* genomes (7–15). The programs RNAmmer (16), tRNAscan-SE (17), and Prodigal (18) were used for gene prediction. The gene names and product descriptions were first annotated based on the homologous genes in other *Spiroplasma* genomes, as identified by OrthoMCL (19). Subsequent manual curation was based on BLASTp (20) searches against the NCBI nonredundant database (21) and the KEGG database (22, 23). Putative clustered regularly interspaced short palindromic repeats (CRISPRs) were identified using CRISPRFinder (24).

Our resequencing analysis identified 13 polymorphic sites, including 12 single-nucleotide polymorphisms and one 1-bp indel in a homopolymeric region. It is unclear if these polymorphisms reflect true genetic variations or are artifacts of the sequencing technologies used. After correcting for these polymorphisms, the *S. turonicum* Tab4c^T^ chromosome described in this work is 1,261,375 bp in size and has a G+C content of 24.2%. The two *S. turonicum* genomes both have one set of 16S-23S-5S rRNA genes, 29 tRNA genes (covering all 20 amino acids), and one 2,940-bp CRISPR locus (containing 44 spacers). However, the annotation of protein-coding genes differs between the two genomes. In CP012328.1, the annotation includes 1,085 protein-coding genes and no pseudogenes. Several of these predicted protein-coding genes appeared to be fragments of disrupted open reading frames and were merged into pseudogenes in our annotation. In the first version of our annotation, the *S. turonicum* Tab4c^T^ genome contains 1,066 protein-coding genes and eight pseudogenes. Finally, the annotation of gene name and product description in this newly reported *S. turonicum* genome is more consistent with the majority of published *Spiroplasma* genomes (7–15).

### Accession number(s).

The complete genome sequence of *S. turonicum* Tab4c^T^ has been deposited at DDBJ/EMBL/GenBank under the accession number CP013860.
